# Editorial: Women in pediatric and adolescent sleep, volume II

**DOI:** 10.3389/frsle.2025.1577860

**Published:** 2025-04-04

**Authors:** Joanna E. MacLean, Sejal Jain

**Affiliations:** ^1^Department of Pediatrics, University of Alberta, Edmonton, AB, Canada; ^2^Women and Children's Health Research Institute, Faculty of Medicine and Dentistry, University of Alberta, Edmonton, AB, Canada; ^3^Stollery Children's Hospital, Edmonton, AB, Canada; ^4^UT Southwestern Medical Center, Dallas, TX, United States

**Keywords:** pay gap, salary and compensation, sex, gender, women

In volume II of “*Women in pediatric and adolescent sleep*,” we highlight the diverse work of women researchers working in the area of pediatric and adolescent sleep. The seven articles in this volume are from a research area inspired, started, or sparked by women; are studies led by women; or celebrate outstanding female researchers and their contributions to pediatric and adolescent sleep.

Women likely make up the minority of sleep researchers, although this may be improving. In a 2021 study from two national U.S. sleep societies, the American Academy of Sleep Medicine (AASM) and the Sleep Research Society (SRS), women made up only 17% of 164 U.S.-based AASM/SRS award recipients over 40 years (Naime and Karroum, 2021). An analysis of the trend over time did show an increase from 0% of award recipients being women in the 1980s' to 32% of recipients being women in the 2010s. U.S.-based women physicians received proportionally fewer AASM/SRS awards than women non-physician PhDs (10% vs. 29%). A 2024 study, using data from the AASM Diversity, Equity and Inclusion annual reports, showed that women's membership in the AASM increased from 33% in 2019 to 39% in 2022 (Shawa and Ehsan, 2024). Women's representation on the AASM Foundation's board of directors also increased from 20% for 1998 to 2017 to 50% for 2017 to 2022. Over its 50-year history, only 18% of all AASM presidents were women; the most recent 10 years, however, do show an upward trend, with women comprising 40% of AASM presidents and 80% of AASM Foundations presidents in the last 5 years (2018–2022). While these studies do not specifically look at the representation of women researchers in sleep, these data suggest that there has been progress in the number of leadership positions held by women in sleep medicine professional organizations.

Despite the improving trends of women's representation in membership, leadership, and awards, compensation inequities between women and men continue. The 2024 AASM study showed that the salaries of women physicians are 19% (approximately $62,000 USD) less than their male counterparts (Shawa and Ehsan, 2024). Differences are still seen when calculating compensation per work relative value unit (12% lower for women) or for total benefits (15% lower for women). These gender-related wage gaps are not unique to sleep physicians or U.S.-based physicians or researchers. Results from the analysis of publicly available Canadian data show that women physicians made up < 35% of 10 specialties with the highest gross and net income (Cohen and Kiran, 2020). By contrast, women accounted for 47%, 48%, and 62% of the specialties with the lowest income (family medicine, psychiatry, and pediatrics, respectively). Differences in hours of work are not sufficient to explain this gap. A national survey on the gender pay gap of the National Health Services (NHS; publicly funded health services) in the United Kingdom was 22%, a gap that is three times larger than for private-sector physicians or other public-sector health professionals (Jones and Kaya, 2024). Discrepancies in the pay gap between males and females may be attributable, at least in part, to differences in starting salaries (Catenaccio et al., 2022) and supplemental income (Miller et al., 2022), which are not explained by specialty, academic rank, work hours, research time, and other factors (Jagsi et al., 2013). Over the course of a career, this difference in wages adds up, with one study estimating that, on average, female U.S. physicians earn $2 million USD less than male physicians across a 40-year career (Whaley et al., 2021). An analysis of data from the Surveys of Doctorate Recipients conducted by the National Science Foundation shows pay gaps between men and women science and engineering doctorate holders working in both academia and industry (Ding et al., 2021). For the 1995–2017 period, women earned 5.3% less than men in academia, compared to 3.5% less in industry. This gap was similar for the 1995–2003 and 2006–2017 periods. The pay gap evolves differently across sectors, with equal pay for men and women starting in academia but widening over the career span. In contrast, men earn more to start within in industry, with the wage gap narrowing across the career span. Reducing and removing these inexplicable inequities in salary and compensation is important to attract women physicians and researchers to pediatric and adolescent sleep, as well as to research careers more broadly.

The topics covered in this Research Topic are diverse, showing the breadth and depth of research in pediatric and adolescent sleep. This includes topics spanning a range in age (young children to adolescents), employing a variety of methodological approaches (e.g., mixed methods), focusing on both existing and emerging technologies (e.g., non-invasive ventilation and machine learning), and addressing several pediatric sleep disorders including insomnia and sleep-disordered breathing. A study by Sturludóttir et al. shows the usefulness of machine learning in sleep analysis. Dobson et al. explore the impact of the COVID-19 pandemic on children using non-invasive ventilation using thematic analysis. A mixed-methods analysis of bedtime routines by Papadopoulou et al. highlights both variance in bedtime routines and common barriers to improving bedtime routines. A brief report from Bauducco et al. shows that popularity in adolescent girls comes at the cost of more insomnia symptoms. A systematic review of the relationship between sleep and physical activity by Wang et al. shows that variation in the measurements of sleep parameters and non-standardization of exercise protocols limits the ability to combine data for meta-analysis. Lacki et al. used polysomnography data to explore the characteristic features of children diagnosed with attention-deficit hyperactivity disorder and/or obstructive sleep apnea. Finally, Olmstead et al. used framework analysis to explore children's experience of using long-term non-invasive ventilation.

For additional research from women in sleep, please see these Frontiers Research Topics:

https://www.frontiersin.org/research-topics/59552/women-in-insomnia;
https://www.frontiersin.org/research-topics/59319/women-in-neurology-sleep-disorders;
https://www.frontiersin.org/research-topics/55217/women-in-sleep-and-breathing;
https://www.frontiersin.org/research-topics/47458/women-in-pediatric-and-adolescent-sleep/magazine.
